# Editorial: Unlocking cancer’s secrets: overcoming drug resistance in cancer stem cells

**DOI:** 10.3389/fphar.2026.1913266

**Published:** 2026-07-15

**Authors:** Stefano Martellucci

**Affiliations:** Department of Life Sciences, Health, and Health Professions, Link Campus University, Rome, Italy

**Keywords:** cancer recurrence, cancer stem cells (CSC), CSC sensitization, CSC-mediated resistance, drug resistance, tumor microenvironment

Despite remarkable advances in cancer diagnosis and treatment, therapeutic resistance remains one of the greatest challenges in modern oncology (Holohan et al., 2013; Marine et al., 2020). Tumor recurrence, metastatic dissemination, and treatment failure continue to limit long-term patient survival across a broad range of malignancies. Increasing evidence indicates that these phenomena are driven not only by genetic alterations but also by dynamic cellular states characterized by stemness, plasticity, and adaptive responses to therapeutic pressure (Batlle and Clevers, 2017; Meacham and Morrison, 2013). Cancer stem cells (CSCs), together with other therapy-tolerant cell populations, have emerged as central mediators of these processes, sustaining tumor heterogeneity, promoting tumor evolution, and enabling survival under otherwise lethal conditions (Dean et al., 2005; Zhou et al., 2021). CSCs are characterized by enhanced self-renewal capacity, activation of pro-survival signaling networks, metabolic adaptability, efficient DNA repair mechanisms, and dynamic interactions with the tumor microenvironment, all of which contribute to therapeutic resistance, tumor recurrence, and metastatic progression (Marine et al., 2020; Liu and Wang, 2024).

The Research Topic “*Unlocking Cancer’s Secrets: Overcoming Drug Resistance in Cancer Stem Cells*” was conceived to highlight emerging advances in our understanding of the molecular and cellular mechanisms underpinning CSC-associated resistance and to explore innovative therapeutic strategies to overcome these barriers. Beyond intrinsic cellular programs, interactions between CSCs and the tumor microenvironment critically influence treatment response, metastatic dissemination, and immune escape (Batlle and Clevers, 2017; Prager et al., 2019; Liu and Wang, 2024). The six contributions collected in this Research Topic encompass diverse cancer types, experimental models, and methodological approaches, ranging from comprehensive reviews of CSC biology to original studies that identify biomarkers of treatment response and evaluate novel therapeutic agents targeting stemness-associated pathways.

A recurring theme across several contributions is the remarkable plasticity of resistant tumor cell populations. In their comprehensive review of liver cancer stem cells in hepatocellular carcinoma, Yadav et al. provide an extensive overview of CSC markers, signaling pathways, microenvironmental interactions, and mechanisms responsible for resistance to conventional and emerging therapies. Their work highlights the multifaceted role of liver CSCs in tumor initiation, metastasis, recurrence, and immune evasion, emphasizing the need for therapeutic approaches that specifically target these highly adaptable cell populations.

Complementing this perspective, Lopez-Gonzalez et al. examine the concept of drug-tolerant persister (DTP) cells, a reversible and slow-cycling cellular state increasingly recognized as an early precursor of stable therapeutic resistance. Through a comprehensive scoping review, the authors demonstrate how non-genetic adaptive mechanisms—including epigenetic remodeling, metabolic rewiring, quiescence, and phenotypic plasticity - allow tumor cells to survive treatment and eventually contribute to disease relapse. Importantly, their findings support the view that CSCs and DTPs may represent overlapping manifestations of cellular plasticity operating along a continuum of resistance.

Together, these reviews highlight the increasingly accepted view that therapeutic resistance is not solely driven by a fixed CSC population but rather by dynamic transitions between stem-like and drug-tolerant cellular states.

The identification of predictive biomarkers capable of anticipating therapeutic response remains a critical objective in precision oncology. Addressing this challenge, Zhan et al. employed large-scale genomic analyses and machine-learning approaches to identify a four-gene signature associated with oxaliplatin sensitivity in colorectal cancer. Their findings suggest that molecular profiling may help stratify patients according to treatment responsiveness, thereby improving therapeutic decision-making and reducing unnecessary exposure to ineffective therapies. Such biomarker-driven strategies are particularly relevant in the context of CSC-associated resistance, where intertumoral and intratumoral heterogeneity often complicate clinical management. Importantly, the identified signature may also provide a framework for linking treatment responsiveness to stemness-associated molecular programs, thereby facilitating more personalized therapeutic strategies.

Resistance is also profoundly influenced by interactions between tumor cells and their microenvironment. In this regard, Ramalingam et al. review the role of the CXCR4/CXCL12 signaling axis in lung cancer progression and metastasis. The authors discuss how CXCR4 signaling contributes not only to tumor dissemination but also to immune evasion and therapeutic resistance through its effects on the tumor microenvironment. Their analysis highlights the potential of CXCR4-targeted interventions, particularly the antagonist plerixafor, as promising strategies to disrupt metastatic niches and enhance the efficacy of existing therapies.

The translational dimension of this Research Topic is further strengthened by two original studies that investigate compounds targeting stemness-associated pathways. Guo et al. demonstrate that cyclovirobuxine D, a natural alkaloid derived from Buxus microphylla, suppresses osteosarcoma stemness and promotes apoptosis by modulating the noncanonical NF-κB pathway. Their findings provide additional evidence that targeting signaling networks that govern CSC maintenance may be an effective strategy to limit tumor progression and resistance. Similar observations have been reported in glioblastoma models, where a natural compound reduced glioma-initiating cell stemness, promoted cellular differentiation, suppressed PI3K/Akt/mTOR and STAT3 signaling, and inhibited tumor growth in preclinical models (Colapietro et al., 2020; Colapietro et al., 2022).

Similarly, Gurke Brücksken et al. report that the histone deacetylase inhibitor largazole suppresses stemness-related signaling in triple-negative breast cancer through a miR-125b-5p-dependent downregulation of Musashi proteins. Beyond impairing tumor growth and motility, largazole enhanced radiosensitivity by attenuating DNA repair mechanisms, thereby addressing a fundamental hallmark of treatment-resistant cancer cells. This study illustrates the therapeutic potential of epigenetic modulation to overcome CSC-associated resistance.

Collectively, the contributions assembled in this Research Topic underscore the complexity of therapeutic resistance and highlight the central role of cellular plasticity, stemness, and microenvironmental interactions in shaping cancer evolution and disease recurrence. At the same time, they provide encouraging evidence that these mechanisms can be therapeutically exploited through biomarker-guided approaches, targeted inhibition of resistance pathways, and the development of novel natural and synthetic compounds.

As our understanding of CSC biology continues to evolve, overcoming drug resistance will require therapeutic strategies that target both bulk tumor cells and the adaptive stem-like populations that sustain disease persistence. The studies presented in this Research Topic provide valuable insights into the biological foundations of therapeutic resistance and offer promising avenues for developing more durable and personalized anticancer therapies.
